# Resveratrol attenuates pulmonary fibrosis by inhibiting alveolar epithelial senescence via targeting SASP-related proteins: an integrated bioinformatics-experimental study

**DOI:** 10.3389/fphar.2025.1680998

**Published:** 2025-11-26

**Authors:** Biao Zuo, Su Yuan, Chen Luo, Xu-Qin Du, Yong-Can Wu, Li-Peng Shi, Jin-Xin Chen, Bo-Tao Chen, Jie Zhou, Yi Ren

**Affiliations:** 1 Department of Rehabilitation, Chongqing Traditional Chinese Medicine Hospital, Chongqing, China; 2 Chongqing Key Laboratory of Traditional Chinese Medicine for Prevention and Cure of Metabolic Diseases, Chongqing Medical University, Chongqing, China; 3 Chongqing University of Chinese Medicine, Chongqing, China; 4 Chongqing Traditional Chinese Medicine Hospital, Chongqing, China; 5 Chongqing Jiulongpo Traditional Chinese Medicine Hospital, Chongqing, China

**Keywords:** bleomycin-induced pulmonary fibrosis in mice, resveratrol, alveolar epithelial senescence, bioinformatics, senescence-associated secretory phenotype(SASP)

## Abstract

**Background:**

Pulmonary fibrosis (PF) is a progressive and fatal interstitial lung disease with limited treatment options. Premature senescence of alveolar epithelial type II cells (AT2 cells) plays a critical role in PF pathogenesis. This study aimed to identify natural compounds targeting senescence-related pathways for PF treatment.

**Methods:**

An integrated approach was implemented, combining bioinformatics, artificial intelligence (AI)-assisted molecular docking, ADMET (absorption, distribution, metabolism, excretion, and toxicity) profiling, and experimental validation. Core targets associated with aging-related pulmonary fibrosis (PF) were identified via database mining (GeneCards and AgingAtlas) and protein-protein interaction (PPI) network analysis. Natural compounds were screened using the HERB database, and resveratrol (RES) was selected due to its multi-target activity and favorable ADMET characteristics. The efficacy of RES was evaluated through *in vitro* experiments using bleomycin (BLM)-induced senescent A549 alveolar epithelial cells and *in vivo* studies in a BLM-induced PF mouse model (C57BL/6J). Molecular docking simulations were performed to predict the binding affinity between RES and key targets, including SERPINE1, MMP2, and IL-6.

**Results:**

Bioinformatics identified 322 aging-related PF targets, with TP53, AKT1, STAT3, JUN, and NFKB1 as core regulators. Resveratrol was selected as a top candidate modulating all five core targets and exhibiting optimal drug-likeness. Molecular docking and dynamics simulations confirmed strong binding affinity between RES and key senescence-associated proteins (SERPINE1: −8 kcal/mol; MMP2: −7.5 kcal/mol; IL-6: −7.1 kcal/mol). *In vitro*, RES (10–40 μM) significantly suppressed bleomycin-induced senescence in A549 cells, reducing SA-β-Gal activity and downregulating SERPINE1, MMP2, and IL6 expression. *In vivo*, RES treatment (20–80 mg/kg, 21 days) attenuated bleomycin-induced PF in mice, improving weight loss, reducing alveolar damage, inflammation, and collagen deposition (Masson’s trichrome) in a dose-dependent manner.

**Conclusion:**

Resveratrol effectively inhibits alveolar epithelial cell senescence and ameliorates pulmonary fibrosis, likely by targeting key senescence-associated pathways (e.g., SERPINE1, MMP2, IL-6). This study provides a promising transdisciplinary strategy for anti-fibrotic drug discovery and highlights RES as a potential therapeutic candidate for PF.

## Graphical Abstract

**Figure:**
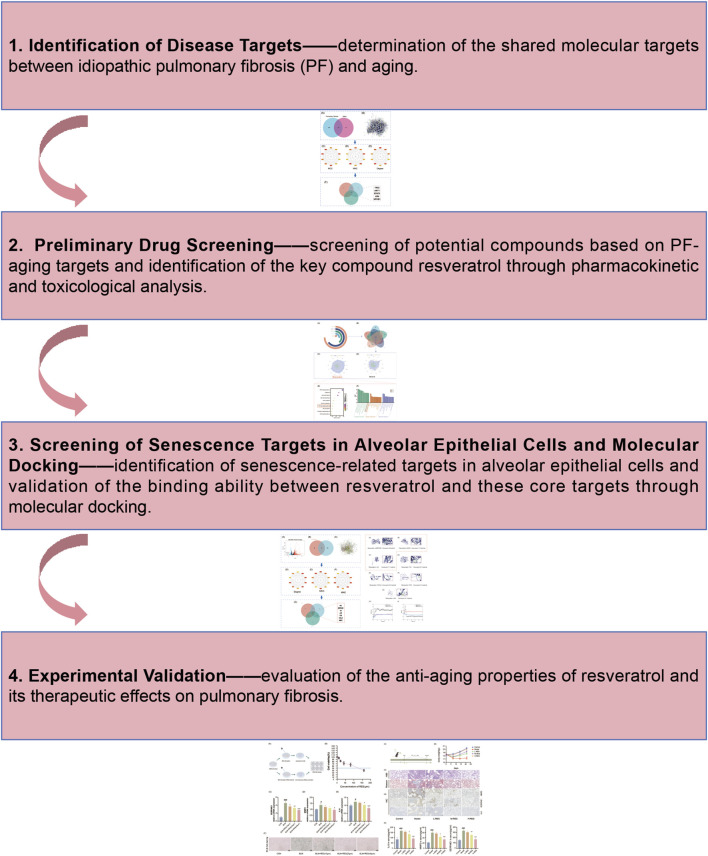

## 1 Introduction

Pulmonary fibrosis (PF) is a progressive, chronic, and often fatal interstitial lung disease characterized by excessive deposition of extracellular matrix (ECM), leading to irreversible scarring and progressive decline in lung function (Richeldi et al., 2017; Koudstaal et al., 2023; Rajan et al., 2023). With a median survival of only 3–5 years after diagnosis, treatment options remain severely limited (Wahsner et al., 2019). Current standard therapies, including anti-fibrotic agents such as pirfenidone and nintedanib, modestly slow disease progression but fail to halt or reverse fibrosis (Lancaster et al., 2017; Finnerty et al., 2021). Moreover, the global incidence of PF is rising—a trend exacerbated by the aftermath of the COVID-19 pandemic and accelerated population aging (George et al., 2020; Patrucco et al., 2023). Thus, developing novel therapeutic strategies for PF represents an urgent priority in respiratory medicine.

Recent advances in the pathogenesis of pulmonary fibrosis have underscored the central role of cellular senescence, particularly premature senescence of alveolar type II epithelial cells (AT2 cells), as a critical driver of disease initiation and progression (Yao et al., 2021; Parimon et al., 2023; Zhu et al., 2024). The alveolar epithelium consists of squamous type I cells (AT1) and cuboidal type II cells (AT2), with AT2 cells acting as progenitor cells responsible for surfactant production and epithelial repair (Chintagari et al., 2016; Guo et al., 2019; Chen and Liu, 2020). Following injury, however, AT2 cells may undergo senescence and adopt a senescence-associated secretory phenotype (SASP), which is marked by sustained secretion of pro-fibrotic mediators such as cytokines, chemokines, and proteases. These factors perpetuate chronic inflammation, promote fibroblast-to-myofibroblast differentiation, and drive pathological ECM remodeling (Birch and Gil, 2020; Maus et al., 2023). Key SASP components, including Serpin Family E Member 1(SERPINE1), matrix metalloproteinase-2 (MMP2), and interleukin-6 (IL-6), are consistently upregulated in PF, disrupting the protease–antiprotease balance and fostering a pro-fibrotic microenvironment (Epstein Shochet et al., 2020; El-Kashef et al., 2022; Chen et al., 2024; Moideen et al., 2024; Sisson et al., 2025). Consequently, targeting alveolar epithelial cell senescence has emerged as a promising therapeutic strategy for mitigating pulmonary fibrosis.

Natural products derived from traditional Chinese medicine (TCM) constitute a valuable resource for anti-fibrotic drug discovery and have demonstrated considerable potential in the treatment of PF (Zhu et al., 2022; Hao et al., 2024). Concurrently, advances in high-throughput sequencing, expansive drug databases, bioinformatics, and artificial intelligence (AI)—particularly AI-driven protein structure prediction tools such as AlphaFold3—have significantly enhanced the accuracy of drug–target interaction predictions and streamlined the drug development process (Fang et al., 2021; Callaway, 2024; Gao et al., 2025). The integration of TCM-derived compounds with contemporary computational approaches thus offers a powerful strategy for identifying novel therapeutics against PF.

In this study, we aimed to develop novel anti-PF agents targeting alveolar epithelial cell senescence via an AI-assisted drug discovery framework. We implemented an integrated computational and experimental strategy, incorporating bioinformatics, molecular docking, molecular dynamics simulations, ADMET (absorption, distribution, metabolism, excretion, toxicity) profiling, and both *in vitro* and *in vivo* validation, to systematically evaluate the therapeutic potential of resveratrol. Our results demonstrate that resveratrol serves as a promising anti-fibrotic candidate by effectively attenuating cellular senescence. Furthermore, this study provides a new conceptual and methodological foundation for AI-enhanced drug development.

## 2 Materials and methods

### 2.1 Bioinformatic and molecular docking study

#### 2.1.1 Target prediction for pulmonary fibrosis and senescence-related genes

Data were retrieved from multiple public repositories on December 11, 2024. The resources interrogated included GeneCards (Version 5.25; http://www.genecards.org) and the Aging Atlas database (last updated in May 2024; https://ngdc.cncb.ac.cn/aging/index).

Pulmonary fibrosis (PF)-related genes were identified from the GeneCards database (Version 5.25; http://www.genecards.org) using “Idiopathic Pulmonary Fibrosis” as the search query (Stelzer et al., 2016). Genes with a Relevance Score >5 were selected as potential PF-related targets. Senescence-associated genes were obtained from the Aging Atlas database (update date: 2024-05; https://ngdc.cncb.ac.cn/aging/index), filtered for *Homo sapiens* entries. The overlapping genes between PF-related and senescence-related gene sets were subsequently identified as common molecular targets (Aging Atlas Consortium, 2021).

#### 2.1.2 Protein-protein interaction network and critical subnetwork construction

The STRING database (version 12.0) was used to construct a protein-protein interaction (PPI) network (Szklarczyk et al., 2023). The database was accessed on February 16, 2025, via the official website (https://cn.string-db.org/), with a minimum interaction score threshold set to 0.7. After parameter standardization, the resulting PPI network was imported into Cytoscape (version 3.10.3) for topological analysis (Shannon et al., 2003). Hub proteins within the network were systematically identified using Cytohubba (Chin et al., 2014), a plugin for Cytoscape, based on three algorithmic ranking methods: MNC, MMC, and Degree. Genes ranked among the top 10 across all three algorithms were selected to construct a subnetwork. Consensus hub genes were subsequently identified through intersection analysis of these ranked lists.

#### 2.1.3 Natural product screening, pharmacokinetic and toxicological profiling

Consensus hub targets, identified through intersection analysis, were systematically screened using the HERB database (version 2.0; http://herb.ac.cn/) (Gao et al., 2025) to retrieve pharmacologically active natural compounds associated with these targets. The database was queried on December 11, 2024. Candidate compounds demonstrating multi-target activity were prioritized based on their target congruence scores. Those exhibiting the highest target overlap coefficients were selected as core small-molecule candidates for further analysis.

The chemical structures of these candidate natural compounds were obtained from the PubChem database (https://pubchem.ncbi.nlm.nih.gov/) (Kim et al., 2023). Predictive ADMET (Absorption, Distribution, Metabolism, Excretion, and Toxicity) profiling was then performed using the ADMETLab 3.0 platform (https://admetmesh.scbdd.com/) (Fu et al., 2024).

#### 2.1.4 KEGG pathway enrichment and GO enrichment analysis

To systematically elucidate the polypharmacological mechanisms of resveratrol, this study first retrieved related high-throughput experimental data from the HERB database (http://herb.ac.cn/). The specific search parameters were as follows: ingredient name “resveratrol” (ID: HBIN042111), species “*Homo sapiens*,” with all up- and downregulated genes displayed (database access date: December 11, 2024). Based on the potential targets obtained from this screening, functional enrichment analysis was further performed, including Kyoto Encyclopedia of Genes and Genomes (KEGG) pathway enrichment and Gene Ontology (GO) biological process enrichment. For result visualization, only the top 10 terms meeting the significance threshold (P < 0.05) and ranked by enrichment degree were displayed to highlight the most statistically significant biological findings.

#### 2.1.5 Identification of senescence-associated genes in alveolar epithelial cells

Senescence-associated differentially expressed genes (DEGs) in A549 alveolar epithelial cells were identified using the GSE155941 dataset from the GEO database (https://www.ncbi.nlm.nih.gov/geo) (Barrett et al., 2013; Sullivan et al., 2021). The dataset was analyzed with R software (version 4.5.0) and the “limma” package (Ritchie et al., 2015) to identify DEGs between normal and senescence-induced A549 cells under the following thresholds: adjusted P-value <0.05 and |log2 FC(fold change)| > 1.5. Differential expression patterns were visualized via volcano plot, and statistically significant DEGs were annotated for subsequent functional analyses.

#### 2.1.6 Molecular docking

Protein structures of core senescence-associated targets were retrieved from the AlphaFold3 artificial intelligence platform (updates: march 2025, https://alphafold.com) (Callaway, 2024). Based on hub gene prioritization results, molecular docking simulations between resveratrol and these core alveolar epithelial senescence proteins were performed using the CBDock2 computational tool (https://cadd.labshare.cn/cb-dock2/index.php) (Liu et al., 2022).

#### 2.1.7 Molecular dynamics (MD) simulation

MD simulations were carried out using GROMACS 2020.3 to investigate the interactions between the receptor and ligand. The amber99sb-ildn force field and the general Amber force field (GAFF) were applied for protein and ligand parameters, respectively. A simulation box was constructed with a minimum distance of 1.0 nm between the protein and box boundaries. The system was solvated using the SPC216 water model and neutralized with Na^+^ and Cl^−^ ions. Energy minimization was performed via the steepest descent algorithm to remove steric clashes. Pre-equilibrium was conducted under NVT and NPT ensembles for 100 ps each at 300 K and 1 bar. Subsequently, a 100 ns production MD simulation was run under periodic boundary conditions, with temperature and pressure maintained at 300 K and 1 bar using the V-rescale and Parrinello-Rahman methods, respectively. The leapfrog integrator with a 2 fs time step was employed to solve the equations of motion. Long-range electrostatics were treated with the Particle Mesh Ewald (PME) method (Fourier spacing = 0.16 nm), and bond constraints were applied with LINCS. Trajectory analysis and visualization were performed using VMD 1.9.3 and PyMOL 2.4.1 (https://pymol.org/). The binding free energy was estimated using gmx_MMPBSA (http://jerkwin.github.io/gmxtool).

### 2.2 Drugs and reagents

Bleomycin (Zhejiang Hisun Pharmaceutical Co., Ltd., Hangzhou, China; CAS No.: 11056-06–7) and resveratrol (purity ≥99%; MedChemExpress, Shanghai, China; Catalog No.: HY-16561) were used in this study. The following antibodies were employed: anti-MMP2 (Abcam, catalog # ab86607), anti-SERPINE1 (Abcam, catalog # ab317604), and anti-IL-6 (Abcam, catalog # ab290735), all obtained from Abcam (Waltham, MA, United States). Carboxymethylcellulose (CMC; CAS No.: CC0113) was purchased from Leagene Biotechnology (Beijing, China). Commercial assay kits were acquired as follows: cytokine quantification kits for SERPINE1 (catalog # QZ-11830), IL-6 (catalog # QZ-10260), and MMP2 (catalog # QZ-15890) from Jiubang Biotech (Xiamen, China); Senescence Detection Kit (SA-β-Gal; catalog #C0602) and Cell Counting Kit-8 (CCK-8; catalog #C0037) from Beyotime Biotech (Shanghai, China).

### 2.3 *In vitro* study

#### 2.3.1 Cell culture and treatment

A549 cells (Shanghai Cell Bank, Chinese Academy of Sciences) were maintained in DMEM (Gibco) supplemented with 10% fetal bovine serum (Biological Industries) and 1% penicillin-streptomycin (Solarbio) at 37 °C in a 5% CO2 humidified atmosphere.

#### 2.3.2 Cell viability

A549 cells (5 × 10^3^/well) were seeded in 96-well plates and treated with graded concentrations of Res for 72 h. After adding 10 μL CCK-8 reagent, plates were incubated at 4 °C for 90 min. Absorbance (450 nm) was quantified using a Biotek microplate reader, with cell viability normalized to untreated controls.

#### 2.3.3 Real-time quantitative PCR analysis (RT-qPCR)

The mRNA expression levels of IL6, MMP2, and SERPINE1, along with the reference gene GAPDH, were measured using real-time quantitative polymerase chain reaction (RT-qPCR). Total RNA was extracted from A549 cells with RNAiso Plus reagent (Takara Bio Inc., Kusatsu, Japan; Cat. # 9109) following the manufacturer’s protocol. Briefly, cells were seeded in 6-well plates at a density of 3 × 10^5^ cells per well and cultured for 24 h before treatment. After treatment, the cells were washed twice with cold Phosphate-Buffered Saline (PBS) and lysed directly in the well using 1 mL of RNAiso Plus reagent. RNA concentration and purity were determined using a NanoDrop 2000 spectrophotometer (Thermo Fisher Scientific, Waltham, MA, United States). All samples exhibited A260/A280 ratios between 1.9 and 2.1, confirming high RNA purity without protein contamination.

Complementary DNA (cDNA) was synthesized from 1 μg of total RNA using the Evo M-MLV RT Mix Kit (Accurate Biology, Hunan, China; Cat. # AG11728) in a 20 μL reaction volume. Genomic DNA was eliminated by treatment with gDNA Eraser at 42 °C for 2 min. The reverse transcription reaction was performed at 37 °C for 15 min, followed by enzyme inactivation at 85 °C for 5 s. Quantitative PCR was carried out using the SYBR Green Premix Pro Taq HS qPCR Kit (Accurate Biology, Hunan, China; Cat. # AG11701) on a CFX96 Touch Real-Time PCR Detection System (Bio-Rad Laboratories, Hercules, CA, United States). Each 20 μL reaction mixture contained 10 μL of SYBR Green Master Mix, 0.8 μL of each forward and reverse primer (10 μM), 2 μL of diluted cDNA template (1:10), and 6.4 μL of nuclease-free water. The thermal cycling protocol consisted of an initial denaturation at 95 °C for 30 s, followed by 40 cycles of 95 °C for 5 s and 60 °C for 30 s. Melting curve analysis was performed by heating from 65 °C to 95 °C at a rate of 0.5 °C per second to confirm amplification specificity and the absence of primer dimers. All samples were run in technical triplicates, and both no-template controls (NTCs) and no-reverse transcription controls (NRTs) were included in each experiment to monitor for potential contamination.

Gene expression was analyzed using the 2^−ΔΔct^ method (Livak and Schmittgen, 2001). The primer sequences used are listed in Table 1.

**TABLE 1:** Primer sequences used for RT-qPCR.

Primers	Forward	Reverse
IL6	CAC​TGG​TCT​TTT​GGA​GTT​TGA​G	GGA​CTT​TTG​TAC​TCA​TCT​GCA​C
MMP2	CCT​GCA​AGT​TTC​CAT​TCC​GC	CTT​CTT​GTC​GCG​GTC​GTA​GT
SERPINE1	GCA​CCA​CAG​ACG​CGA​TCT​T	ACC​TCT​GAA​AAG​TCC​ACT​TGC
GAPDH	GGC​ATG​GAC​TGT​GGT​CAT​GAG	TGC​ACC​ACC​AAC​TGC​TTA​GC

#### 2.3.4 Senescence-associated β-galactosidase (SA-β-gal) staining

A549 cells (2 × 10^5^ per well, 6-well plates) were rinsed twice with PBS, fixed for 15 min at room temperature with 1 mL of 2% (w/v) formaldehyde +0.2% (v/v) glutaraldehyde in PBS, and washed three times (3 min each) with PBS. Cells were overlaid with 1 mL of freshly prepared X-gal working solution (Beyotime, China, cat. C0602: 1 mg/mL 5-bromo-4-chloro-3-indolyl β-D-galactopyranoside, 40 mM citric acid/sodium phosphate pH 6.0, 5 mM K_4_ [Fe(CN)_6_], 5 mM K_3_ [Fe(CN)_6_], 150 mM NaCl, 2 mM MgCl_2_). Plates were sealed with Parafilm to prevent evaporation and incubated for 16 h at 37 °C in a dry oven without CO_2_. Images were acquired on an Olympus IX73 inverted microscope (×10 objective, phase-contrast) by an investigator blinded to treatment; ≥5 non-overlapping fields per well were recorded. SA-β-gal-positive (blue) cells were enumerated with ImageJ v1.54 and expressed as percentage of total cells (≥500 cells per condition).

### 2.4 *In vivo* study

#### 2.4.1 Animals and housing

Male C57BL/6J mice (aged 6–8 weeks, weighing 18 ± 2 g) were obtained from the Laboratory Animal Center of Chongqing Medical University. All animals were housed under specific pathogen-free (SPF) conditions at 22 °C ± 2 °C and 50%–60% relative humidity, with a 12-h light/dark cycle. They were provided with irradiated feed and autoclaved water *ad libitum*. All experimental procedures were reviewed and approved by the Institutional Animal Care and Use Committee of Chongqing Medical University (Approval No. CQMU-20250310) and conducted in accordance with the ARRIVE 2.0 guidelines.

#### 2.4.2 Experimental design

After a 7-day acclimation period, 50 mice were randomly assigned to five experimental groups (n = 10 per group) using a computer-generated randomization sequence as follows.

1. Vehicle control group (received 0.5% w/v sodium carboxymethyl cellulose [CMC-Na] via intragastric administration);
2. BLM model group (received a single intratracheal instillation of bleomycin sulfate at 5 mg/kg in 20 µL sterile saline);
3. BLM + low-dose resveratrol group (20 mg/kg/day, suspended in 0.5% CMC-Na and administered by gavage);
4. BLM + medium-dose resveratrol group (40 mg/kg/day, suspended in 0.5% CMC-Na and administered by gavage);
5. BLM + high-dose resveratrol group (80 mg/kg/day, suspended in 0.5% CMC-Na and administered by gavage).

Based on previous literatureand established modeling protocols from our laboratory, the classic bleomycin-induced pulmonary fibrosis model was employed (Zuo et al., 2022; Yuan et al., 2023; Wang et al., 2025). Briefly, mice were anesthetized via intraperitoneal injection of Avertin (2,2,2-tribromoethanol; Shanghai Duowei Biotechnology Co., Ltd., product no. DW3106) at a dose of 20 μL/g. The trachea was then exposed using a visual laryngoscope (Beijing Yuansen Kaide Biotechnology Co., Ltd., cat. no. HY-LWH03), and bleomycin solution was slowly instilled into the lungs via the trachea at a dose of 5 mg/kg.

The experimental period lasted 22 days. On day 0, pulmonary fibrosis was induced by a single intratracheal instillation of bleomycin. From days 1–21, resveratrol was administered once daily via gavage. Doses of resveratrol were selected based on previous literature and preliminary experiments, and included low- (20 mg/kg), medium- (40 mg/kg), and high-dose (80 mg/kg) groups (Ding et al., 2019; Wang et al., 2024). Body weight was monitored and recorded daily. On day 22, all mice were deeply anesthetized with an oxygen–isoflurane mixture (1.5%–2.5%) and euthanized by exsanguination.

#### 2.4.3 Histopathological analysis

Lung tissue specimens were fixed in 4% paraformaldehyde for 24 h at 4 °C, sequentially dehydrated through graded ethanol series (70%, 80%, 95%, 100%), cleared in xylene, and paraffin-embedded (Leica Biosystems, Germany). Consecutive 4-μm sections were prepared using a Leica RM2235 rotary microtome. Sections underwent: (1) Hematoxylin and eosin (H&E; Aifang Biotechnology, China) staining for histoarchitectural evaluation; (2) Masson’s trichrome (H&E; Aifang Biotechnology, China) staining for collagen deposition analysis. Bright-field imaging was performed with an Olympus BX53 microscope (DP74 camera, ×40 objective; Japan).

#### 2.4.4 Immunohistochemical staining

Deparaffinized sections underwent antigen retrieval in citrate buffer (pH 6.0, 95 °C, 20 min). Following sequential incubation with primary antibodies (4 °C/overnight) and species-specific HRP-conjugated secondary antibodies (37 °C/1 h), immunostaining was developed using DAB chromogen with hematoxylin counterstaining for microscopic evaluation.

#### 2.4.5 Enzyme-linked immunosorbent assay (ELISA)

Serum concentrations of matrix metalloproteinase-2 (MMP2), interleukin-6 (IL-6), and plasminogen activator inhibitor-1 (SERPINE1) in mice were determined using commercial sandwich ELISA kits (Jiubang Biotechnology, Fujian, China) according to the manufacturer’s protocols. All assays were performed in duplicate with appropriate controls included.

### 2.5 Statistical analysis

All statistical analyses were performed using GraphPad Prism version 8.0 (GraphPad Software, San Diego, CA, United States). Continuous data are expressed as mean ± standard deviation (SEM). For comparisons among multiple groups, one-way analysis of variance (ANOVA) was applied after confirming normality (Shapiro-Wilk test) and homogeneity of variance (Brown-Forsythe test). When ANOVA indicated significant differences (p < 0.05), Tukey’s honestly significant difference (HSD) *post hoc* test was used for pairwise comparisons. Statistical significance was defined as two-tailed p < 0.05.

## 3 Results

### 3.1 Identification of aging-related genes in pulmonary fibrosis

**FIGURE 1:**
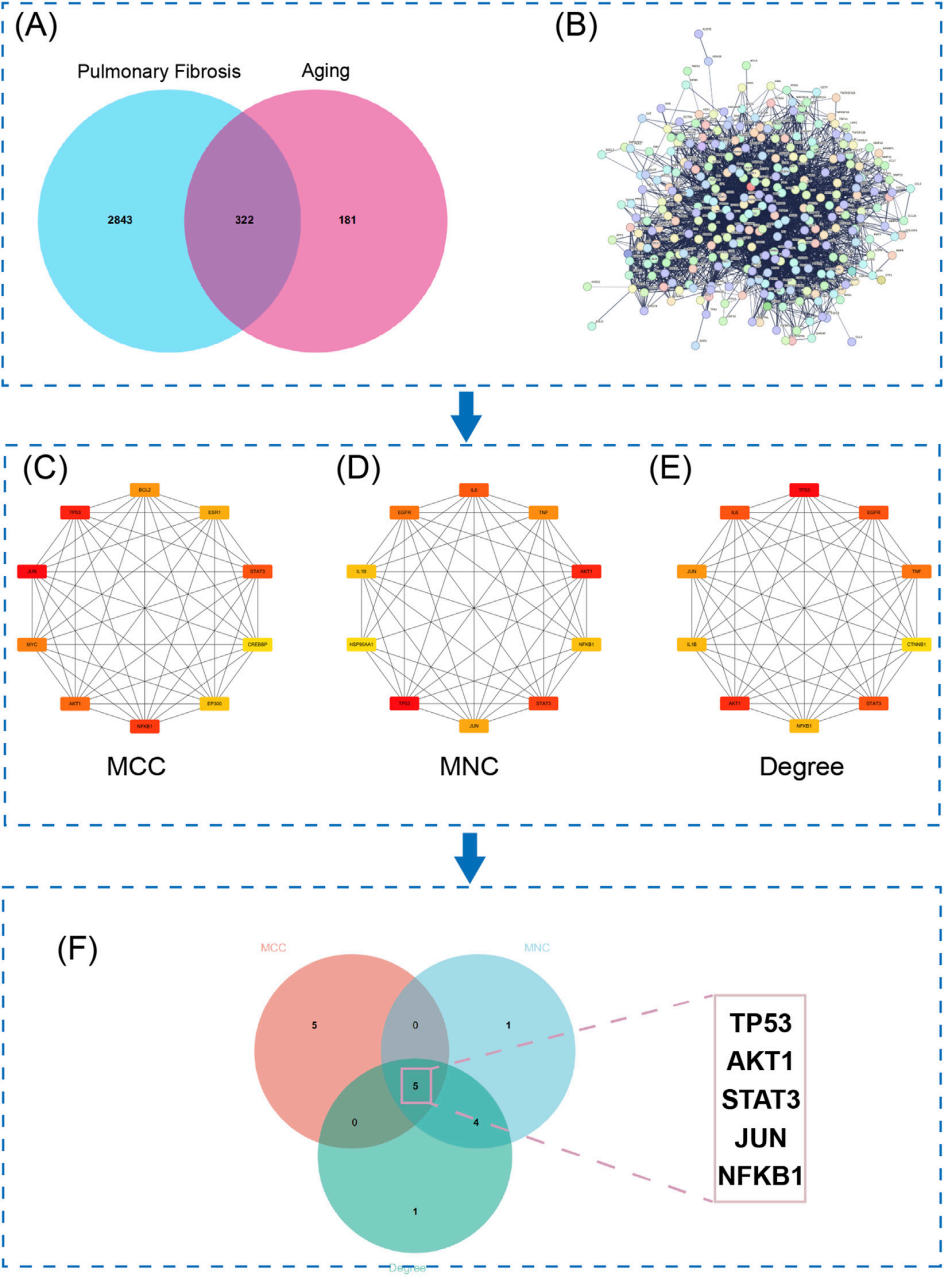
Identification of five hub proteins shared between pulmonary fibrosis and aging. **(A)** Venn diagram showing the overlap of proteins associated with pulmonary fibrosis (PF) and aging, with 322 common proteins identified. **(B)** Protein-protein interaction (PPI) network constructed from the 322 overlapping proteins derived from PF and aging-related datasets. **(C–E)** The top 10 hub proteins ranked by the Maximal Clique Centrality (MCC), Maximum Neighborhood Component (MNC), and Degree algorithms, respectively. **(F)** Five core hub proteins consistently identified by all three algorithmic methods.

To identify genes implicated in aging-related pulmonary fibrosis (PF), we retrieved 3,165 PF-associated genes from the GeneCards database and 503 aging-related genes from the AgingAtlas database. Venn diagram analysis revealed 322 overlapping genes, indicating their potential involvement in the pathogenesis of age-related PF (Figure 1A). Detailed results are provided in Supplementary Table S1.

A protein–protein interaction (PPI) network was constructed using the STRING database (Figure 1B). Core regulatory genes were identified via Cytoscape using three topological algorithms (MCC, MNC, and Degree), with the top 10 genes from each method selected (Figures 1C–E). Intersection analysis yielded five core genes: TP53, AKT1, STAT3, JUN, and NFKB1 (Figure 1F), suggesting their central role in PF-related senescence. Complete analytical results are available in Supplementary Tables S2–S5.

### 3.2 Preliminary screening of natural drug candidates

**FIGURE 2:**
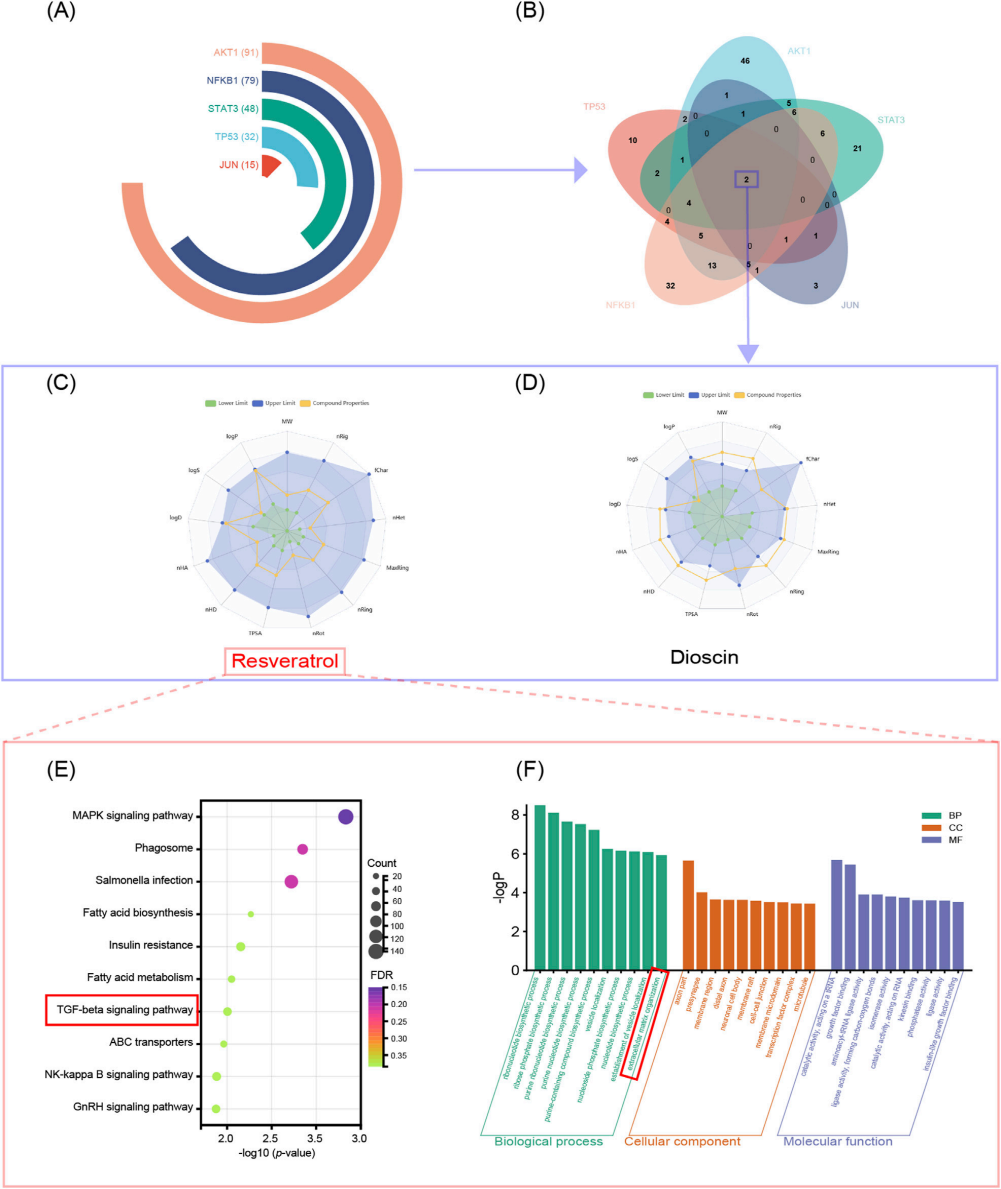
Drug screening based on shared senescence–PF targets and identification of resveratrol as a candidate compound. **(A,B)** Multi-target compound screening was performed using drug databases and the five hub targets common to both cellular senescence and pulmonary fibrosis (PF). Venn diagram analysis identified two compounds, resveratrol and dioscin, capable of acting on all five targets. **(C,D)** Comprehensive ADMET (absorption, distribution, metabolism, excretion, toxicity) profiling showed that resveratrol possesses more favorable pharmacokinetic and safety properties than dioscin, supporting its selection as the lead candidate. **(E,F)** GO and KEGG enrichment analyses indicate that the anti-fibrotic effects of resveratrol are mediated through regulation of the TGF-β1 signaling pathway (a central driver of PF) and suppression of aberrant extracellular matrix (ECM) deposition (a hallmark pathological feature of PF).

Based on the identified core genes, we queried the Herb database to screen for potential natural small-molecule drugs. Initial screening showed that the core genes JUN, NFKB1, STAT3, TP53, and AKT1 corresponded to 15, 79, 48, 32, and 91 candidate compounds, respectively (Figure 2A, see Supplementary Table S6). Intersection analysis revealed that both resveratrol and dioscin were capable of simultaneously targeting all five core genes (Figure 2B).

Subsequent pharmacokinetic evaluation using ADMETLAB 3.0 indicated that resveratrol satisfied all fundamental criteria, whereas dioscin exceeded the acceptable limits for molecular weight (MW), number of hydrogen bond acceptors (nHA), and donors (nHD) (Figures 2C,D). Resveratrol exhibited favorable drug-like properties, including a quantitative estimate of drug-likeness (QED) score of 0.692, and complied with both Lipinski’s and Pfizer’s rules. It demonstrated good intestinal absorption, with a Caco-2 permeability value of −4.917, a plasma protein binding (PPB) rate of 88.573%, and a low blood-brain barrier (BBB) penetration value (0.003), suggesting a high therapeutic index with minimal central nervous system exposure. Regarding excretion, resveratrol showed a plasma clearance (CLplasma) of 9.035 mL/min/kg and a half-life (T_1_/_2_) of 1.456 h, indicating moderate clearance and a short half-life. Metabolic profiling suggested that resveratrol does not inhibit CYP2C19, supporting its unaltered metabolic processing. Toxicity assessments indicated a low risk of hERG inhibition, drug-induced liver injury (DILI), rat oral acute toxicity, and nephrotoxicity (see Supplementary Materials 1,2).

Furthermore, KEGG pathway enrichment analysis revealed that resveratrol significantly modulates the TGF-β1 signaling pathway—a central regulator in PF pathogenesis (Figure 2E). GO enrichment analysis indicated a strong association between resveratrol and extracellular matrix (ECM) formation, a process critically involved in PF pathology (Figure 2F).

Based on these comprehensive ADMET properties and mechanistic enrichment analyses, resveratrol was selected for further experimental evaluation.

### 3.3 Identification of alveolar epithelial senescence-related pulmonary fibrosis target genes

**FIGURE 3:**
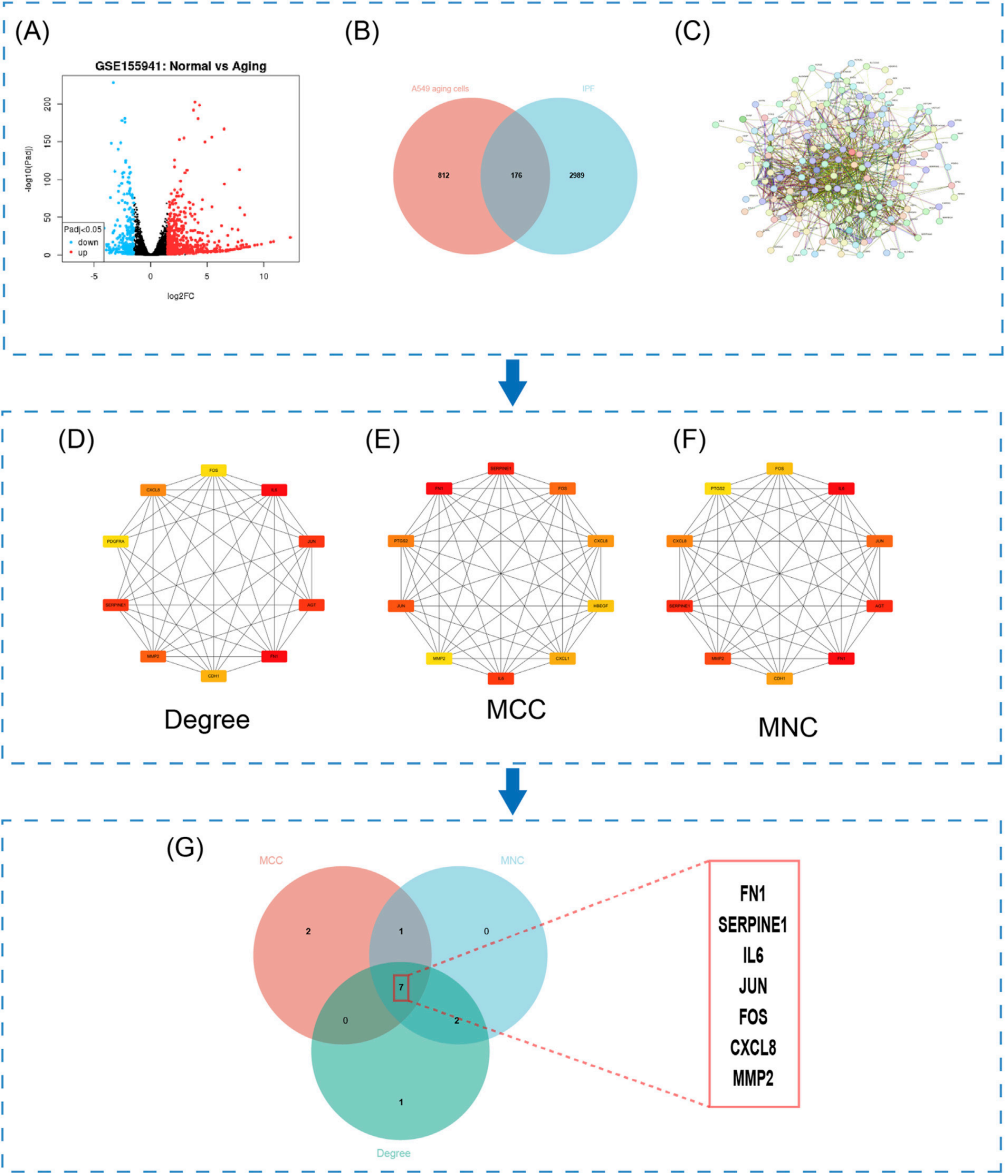
Identification of seven hub proteins associated with epithelial cell senescence in PF. **(A)** Senescence-associated proteins identified in alveolar epithelial cells (A549). **(B)** A total of 176 overlapping proteins associated with both PF and A549 cellular senescence. **(C)** Protein–protein interaction (PPI) network constructed from these 176 overlapping proteins. **(D–F)** Top 10 hub proteins identified using the Degree, Maximal Clique Centrality (MCC), and Maximum Neighborhood Component (MNC) algorithms, respectively. **(G)** Venn diagram illustrating the seven overlapping hub proteins commonly identified by the MCC, MNC, and Degree algorithms.

To identify genes regulating idiopathic pulmonary fibrosis (PF) via alveolar epithelial cell senescence, we obtained transcriptomic data from normal and senescent human alveolar epithelial A549 cells from the GEO database (accession GSE155941). A total of 988 senescence-associated differentially expressed genes (DEGs) were identified in epithelial cells (Figure 3A). Intersection analysis between these DEGs and 3,165 known PF-related genes revealed 176 overlapping genes, suggesting their potential involvement in epithelial senescence-mediated PF (Figure 3B; see Supplementary Table S7 for details). These overlapping genes were used to construct a protein–protein interaction (PPI) network using the STRING database for further identification of key targets (Figure 3C). Topological and intersection analyses were performed using the MCC, MNC, and Degree algorithms in Cytoscape (Figures 3D–F; refer to Supplementary Tables S8–S11). Finally, seven core genes were identified: FN1, SERPINE1, IL6, JUN, FOS, CXCL8, and MMP2 (Figure 3G).

### 3.4 Molecular docking and dynamics simulations suggest resveratrol targets senescence markers SERPINE1, IL-6, and MMP2 *in silico*

**FIGURE 4:**
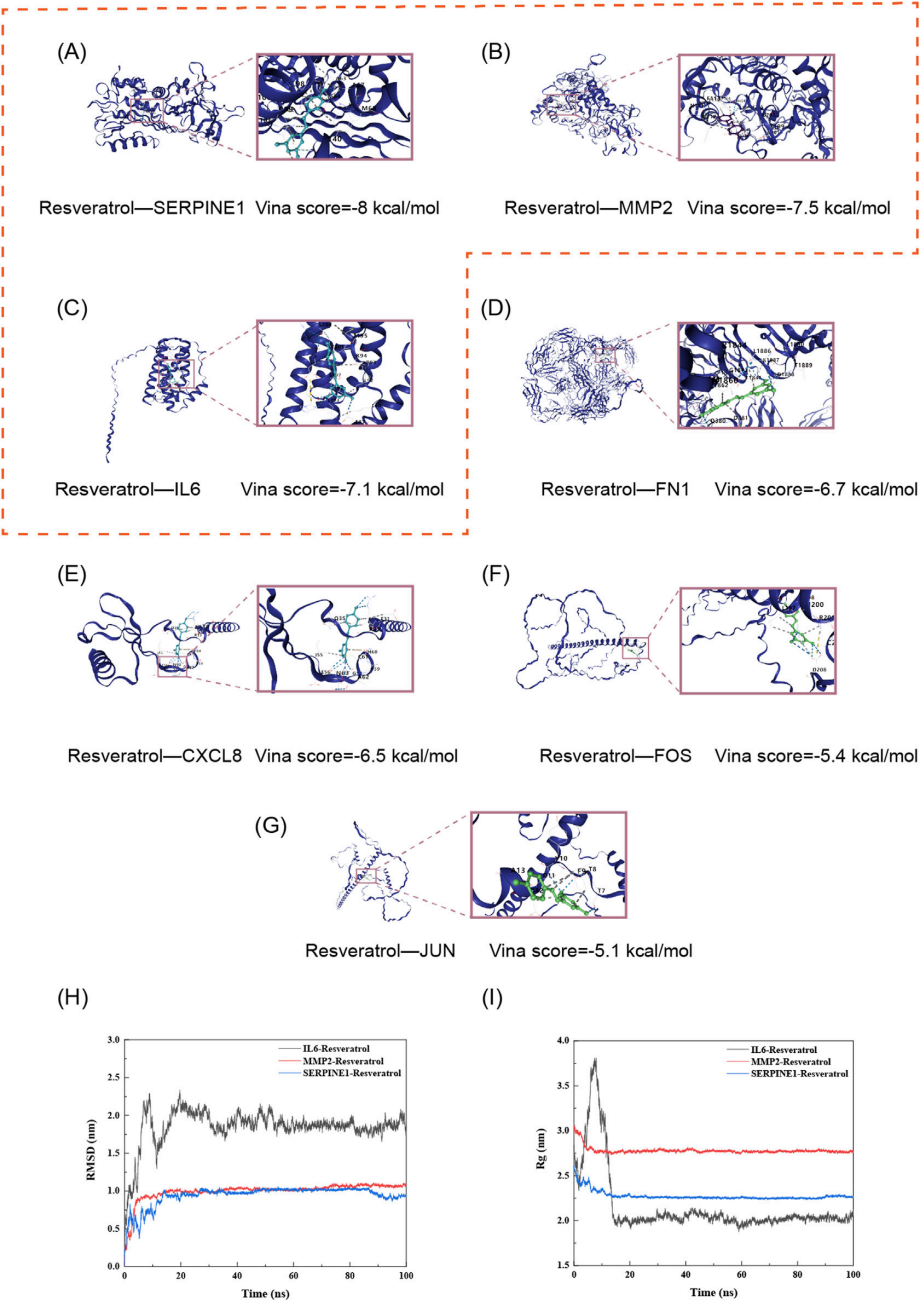
Molecular docking analysis of Resveratrol with potential target proteins. Docking scores (in kcal/mol), calculated using AutoDock Vina, are provided for each complex. More negative values indicate stronger predicted binding affinity. Specific interactions include: **(A)** Resveratrol–SERPINE1 (Score: −8.0 kcal/mol); **(B)** Resveratrol–MMP2 (Score: −7.5 kcal/mol); **(C)** Resveratrol–IL6 (Score: −7.1 kcal/mol); **(D)** Resveratrol–FN1 (Score: −6.7 kcal/mol); **(E)** Resveratrol–CXCL8 (Score: −6.5 kcal/mol); **(F)** Resveratrol–FOS (Score: −5.4 kcal/mol); **(G)** Resveratrol–JUN (Score: −5.1 kcal/mol). **(H)** Root Mean Square Deviation (RMSD): Lower RMSD values and convergence to a stable plateau suggest enhanced stability of the Resveratrol–protein complex. **(I)** Radius of Gyration (Rg): Smaller and less fluctuating Rg values indicate a more compact and stable binding conformation between Resveratrol and the target protein. All scores represent the binding energy of the complex.

Molecular docking is a computational approach widely used to predict the binding affinity and orientation of ligands to receptor proteins. To explore the interactions between resveratrol and key proteins implicated in alveolar epithelial senescence, we performed molecular docking simulations between resveratrol and seven senescence-associated proteins. Based on widely recognized thresholds, a binding energy ≤ −5 kcal/mol indicates substantial binding, while a value ≤ −7 kcal/mol suggests high-affinity binding (Al-Khafaji and Taskin Tok, 2020; Vashisth et al., 2024). Our results revealed that resveratrol exhibits binding potential to all seven senescence-related core proteins (Figure 4). Notably, it displayed high-affinity binding with SERPINE1 (−8.0 kcal/mol), MMP2 (−7.5 kcal/mol), and IL-6 (−7.1 kcal/mol) (Figures 4A–C). These results suggest that resveratrol has strong binding interactions with SERPINE1, MMP2, and IL-6, all of which contribute to alveolar epithelial cell senescence.

Molecular dynamics (MD) simulations provide a more comprehensive and dynamic assessment of ligand–protein complex stability than static molecular docking. Two key metrics, Root Mean Square Deviation (RMSD) and Radius of Gyration (Rg), were employed to evaluate conformational stability. Lower RMSD values that converge to a stable plateau indicate a well-balanced and stable complex. Similarly, reduced and steady Rg values are indicative of a compact and stable protein–ligand conformation. As shown in Figure 4H, the RMSD trajectories of the IL-6–resveratrol, MMP2–resveratrol, and SERPINE1–resveratrol complexes all reached equilibrium after 20 ns, confirming the stability and reliability of the simulation process. The average RMSD values were 1.8587 ± 0.2448 nm for IL-6–resveratrol, 0.9978 ± 0.1232 nm for MMP2–resveratrol, and 0.9419 ± 0.1321 nm for SERPINE1–resveratrol, indicating robust binding stability. As presented in Figure 4I, the Radius of Gyration (Rg) was used to evaluate the compactness of the protein structures during simulation, reflecting the distance distribution of atoms from the centroid. A decline in Rg values was observed during the simulations for the resveratrol-bound complexes of IL-6, MMP2, and SERPINE1, implying that resveratrol enhances their structural compactness upon binding.

Overall, these results demonstrate that resveratrol forms stable conformational complexes with SERPINE1, IL-6, and MMP2, implying its potential role in mitigating alveolar epithelial cell senescence through targeted protein interactions.

### 3.5 Resveratrol inhibits bleomycin-induced senescence in alveolar epithelial cells

**FIGURE 5:**
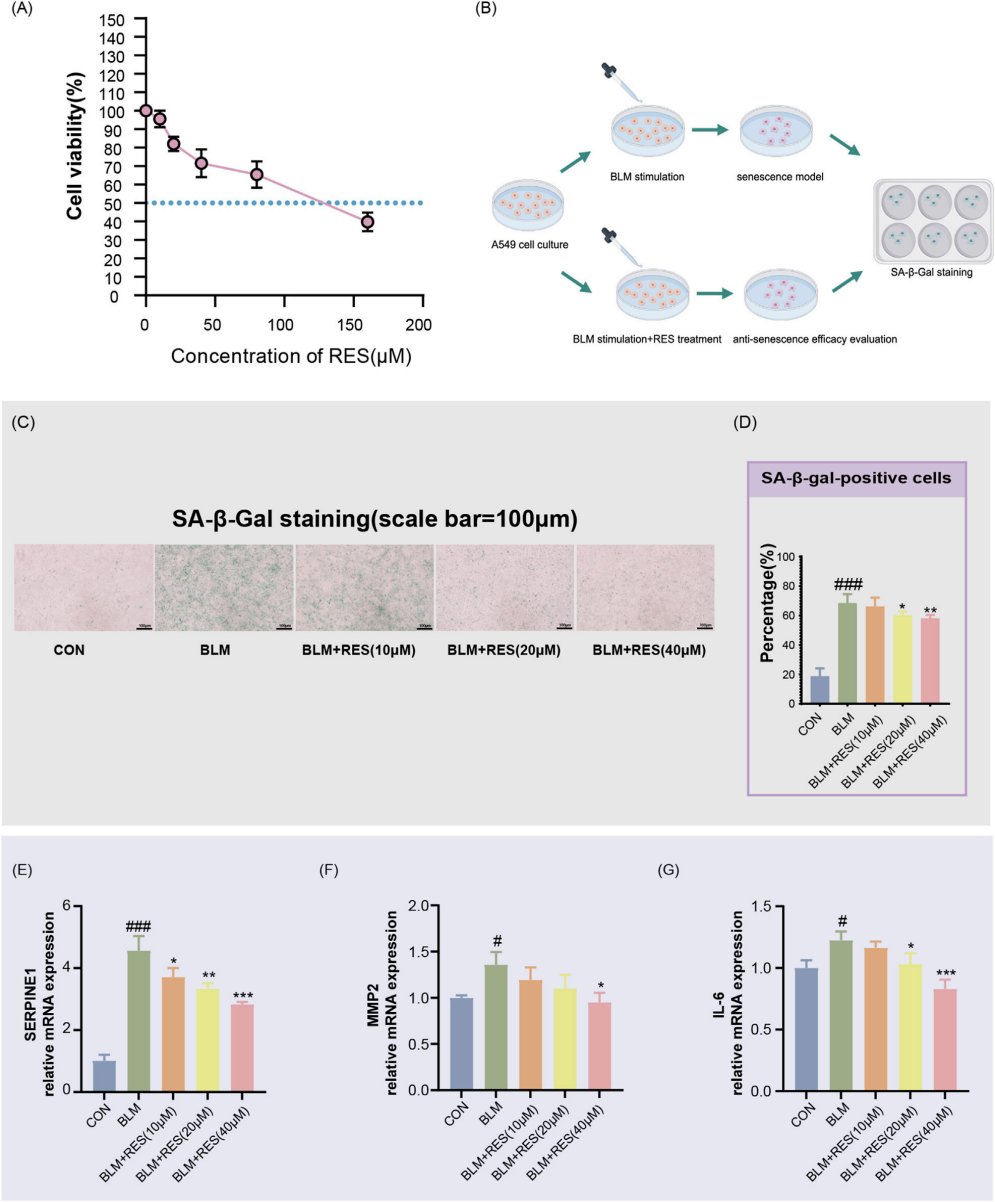
Resveratrol alleviates cellular senescence in A549 cells. **(A)** Cell viability was measured using the CCK-8 assay to determine the optimal concentration of RES (0, 20, and 40 μM). **(B)** Schematic illustration of the experimental design. Cellular senescence was induced by bleomycin (5 μg/mL), and resveratrol (RES) was administered concomitantly to evaluate its anti-senescence effects. **(C)** Senescence-associated β-galactosidase (SA-β-gal) staining was performed to assess the anti-senescence effect of RES. Senescent cells are characterized by blue staining. **(D)** The percentage of SA-β-gal-positive cells was quantified using ImageJ software by counting 3–5 randomly selected fields per replicate. Data are representative of three independent biological replicates (n = 3). **(E–G)** Effects of RES on the expression of senescence-associated markers (SERPINE1, MMP2, and IL-6) (n = 6). Experimental groups: CON (control), BLM (bleomycin-induced senescence), BLM + RES (bleomycin plus resveratrol co-treatment). Data are presented as mean ± SEM. #p < 0.05, ###p < 0.001 vs. Control group; *p < 0.05, **p < 0.01, ***p < 0.001 vs. BLM group. Statistical analysis was performed using one-way ANOVA with Tukey’s honestly significant difference (HSD) *post hoc* test.

The IC_50_ of resveratrol in A549 cells was determined to be 118.1 μM using a CCK-8 assay (Figure 5A). Based on this finding, non-cytotoxic concentrations of resveratrol (10, 20, and 40 μM) were selected for further experiments. A cellular senescence model was established by treating A549 cells with 5 μg/mL bleomycin (BLM) for 3 days (Figure 5B).

As shown in Figures 5C,D bleomycin treatment significantly increased the proportion of SA-β-gal-positive cells from 18.65% ± 5.42% to 68.4% ± 6.09% (p < 0.001), without affecting cell viability. Administration of resveratrol at 20 and 40 μM markedly attenuated the bleomycin-induced senescence phenotype, reducing the percentage of SA-β-gal-positive cells to 59.7% ± 2.63% (p < 0.05) and 58.03% ± 2.41% (p < 0.01), respectively, compared to the BLM model group.

Furthermore, bleomycin exposure significantly upregulated the mRNA expression of senescence-associated markers, including SERPINE1 (4.52-fold, p < 0.001), MMP2 (1.36-fold, p < 0.05), and IL6 (1.226-fold, p < 0.05), relative to the untreated controls. Treatment with 40 μM resveratrol effectively reversed these changes, reducing the expression levels to 0.62-fold (p < 0.001), 0.698-fold (p < 0.05), and 0.677-fold (p < 0.05) of those in the model group, respectively (Figures 5E–G).

Together, these data indicate that resveratrol significantly inhibits bleomycin-induced cellular senescence in alveolar epithelial cells.

### 3.6 Resveratrol attenuates senescence-related markers and ameliorates bleomycin-induced pulmonary fibrosis in mice

**FIGURE 6:**
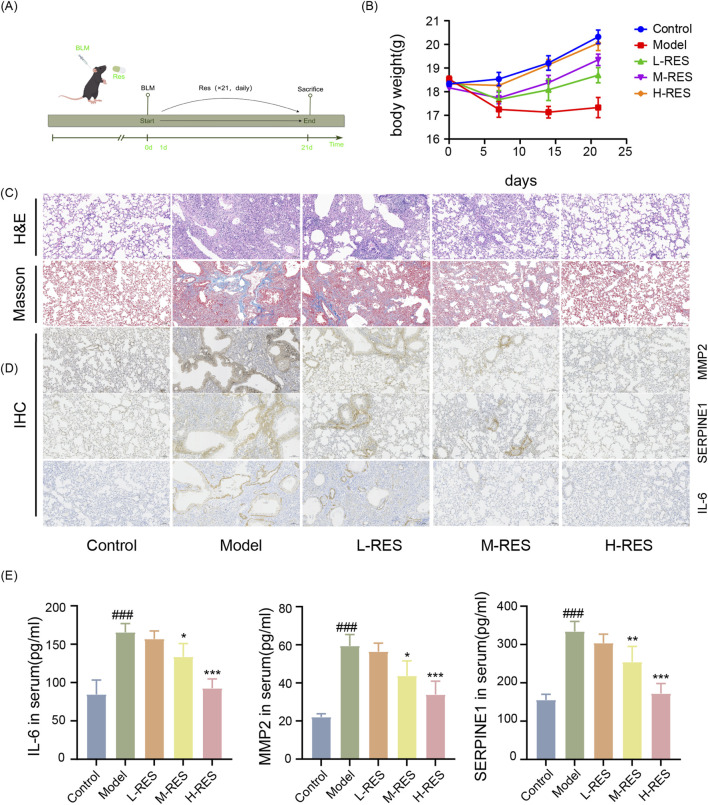
Resveratrol attenuates senescence markers and ameliorates pulmonary fibrosis in mice. **(A)** Experimental timeline. Male C57BL/6 mice (6–8 weeks old) were randomly divided into five groups (n = 10 per group): Control, Model, L-RES (low-dose resveratrol, 20 mg/kg), M-RES (medium-dose, 40 mg/kg), and H-RES (high-dose, 80 mg/kg). Pulmonary fibrosis was induced via oropharyngeal instillation of bleomycin (BLM; 5 mg/kg). RES was administered at the indicated doses. **(B)** Body weight changes over time (n = 6). RES treatment dose-dependently attenuated bleomycin-induced weight loss. **(C,D)** Representative images of lung tissue sections (n = 6). H&E staining revealed improved alveolar architecture; Masson’s trichrome staining showed reduced collagen deposition (blue). Immunohistochemistry (IHC) indicated decreased expression of senescence-associated proteins (SERPINE1, MMP2, IL-6) in RES-treated groups. **(E)** Serum levels of senescence markers (SERPINE1, MMP2, IL-6) as quantified by ELISA (n = 6). RES treatment significantly and dose-dependently reduced these markers. Data are presented as mean ± SEM. ###p < 0.001 vs. Control group; *p < 0.05, **p < 0.01, ***p < 0.001 vs. Model group (one-way ANOVA followed by Tukey’s honestly significant difference (HSD) *post hoc* test.).

We assessed the effects of resveratrol (RES) on bleomycin (BLM)-induced pulmonary fibrosis (PF) in mice (Figure 6A). BLM-treated mice exhibited significant weight loss and delayed recovery compared to the control group (Con). Treatment with low (20 mg/kg, L-RES), medium (40 mg/kg, M-RES), and high (80 mg/kg, H-RES) doses of RES attenuated this weight loss, with the H-RES group showing the most pronounced recovery (Figure 6B).

Histological analysis (H&E staining) revealed normal lung architecture in the Con group. In contrast, the BLM group displayed substantial pathological alterations, including alveolar collapse/narrowing, interstitial thickening, destruction of lung parenchyma, and marked inflammatory cell infiltration (Figure 6C). RES treatment (L-RES, M-RES, H-RES) significantly reduced BLM-induced alveolar structural damage, septal thickening, and inflammatory cell infiltration. These protective effects increased in a dose-dependent manner, with H-RES demonstrating the greatest efficacy. Masson’s trichrome staining confirmed significant collagen deposition, indicative of progressive PF, in BLM-treated mice. RES administration also dose-dependently inhibited BLM-induced collagen deposition, with H-RES showing the strongest effect (Figure 6C).

Immunohistochemical analysis showed elevated expression of senescence-associated genes SERPINE1, MMP2, and IL6 in the BLM group. RES treatment (L-RES, M-RES, H-RES) suppressed the expression of these markers in a dose-dependent manner (Figure 6D). Consistently, ELISA analysis of mouse serum revealed significantly higher levels of SERPINE1, MMP2, and IL6 in the BLM group, which were also suppressed dose-dependently by RES treatment (L-RES, M-RES, H-RES) (Figure 6E).

Collectively, BLM administration induced significant inflammatory and fibrotic pathological changes. RES treatment, particularly at the high dose, significantly ameliorated these alterations.

## 4 Discussion

Currently, anti-senescence strategies are a popular approach in the field of new drug development for pulmonary fibrosis (Zhou and Lagares, 2021). This study innovatively combines the perspectives of organismal aging and alveolar epithelial cell senescence to identify targets for pulmonary fibrosis intervention. Through this perspective, we have selected resveratrol, a natural compound with potential therapeutic prospects. Rigorous evaluations, including pharmacokinetic analysis, molecular docking simulations, and comprehensive validation through *in vitro* and *in vivo* experiments, consistently demonstrated resveratrol’s exceptional anti-senescence and anti-fibrotic properties. These findings not only provide new molecular targets for precision treatment of pulmonary fibrosis but also open up new directions for the development of natural-source drugs. Importantly, these research outcomes hold significant strategic importance for accelerating research progress and new drug development in the field of pulmonary fibrosis.

Cellular senescence is a physiological response to damaging stimuli, characterized by irreversible cell cycle arrest and the secretion of the senescence-associated secretory phenotype (SASP), which includes factors such as SERPINE1, MMP2, and IL-6. Among these, IL-6 serves as a core driver and biomarker of inflammaging and the SASP. SERPINE1 promotes fibrosis and inhibits tissue repair. Matrix metalloproteinase 2 (MMP2) plays a crucial role in extracellular matrix (ECM) remodeling, and its dysregulation can lead to tissue structural damage. Although organismal aging is not entirely synonymous with cellular senescence, the latter constitutes a significant mechanism driving the former. Evidence indicates that senescent type II alveolar epithelial cells contribute to the progression of pulmonary fibrosis (Zhou et al., 2022). Our molecular docking analysis revealed that resveratrol exhibits favorable binding affinity towards these key senescence-associated factors (SERPINE1, MMP2, and IL-6). *In vitro* experiments confirmed that resveratrol alleviates the senescent phenotype in alveolar epithelial cells and significantly suppresses the expression of SERPINE1, MMP2, and IL-6. Furthermore, staining of lung tissue sections validated resveratrol’s inhibitory effect on the expression of these senescence markers. In conclusion, the findings of this study suggest that resveratrol may attenuate pulmonary fibrosis by targeting cellular senescence and the associated secretory phenotype.

This study established an innovative drug development strategy that integrated bioinformatics analysis, ADMET prediction (using ADMETLab 3.0), computational simulations, and experimental validation. Nonetheless, the initial phase relied heavily on bioinformatic predictions for both target identification and drug screening, introducing a degree of inherent uncertainty. Specifically, the identification of core aging-related pulmonary fibrosis genes (TP53, AKT1, STAT3, JUN, NFKB1) and the selection of resveratrol as the lead compound were based on database mining (e.g., Genecards, AgingAtlas, String, Herb). While this approach provided a useful starting point, subsequent experimental validation did not yield the anticipated positive outcomes, suggesting a possible disconnect between predictive models and biological reality.

Although *in silico* ADMET profiling indicated favorable pharmacokinetic properties for resveratrol—including high intestinal absorption—the lack of experimental pharmacokinetic data (e.g., plasma concentration, half-life, metabolic stability) constitutes a major limitation. Consequently, it remains unclear whether the efficacy observed *in vivo* at doses of 20–80 mg/kg reflects sufficient systemic bioavailability or stems from compensation via high dosing due to its known rapid metabolism and elimination. Future studies incorporating integrated pharmacokinetic-pharmacodynamic (PK/PD) modeling will be essential to clarify the exposure-response relationship and guide rational dose optimization.

To ensure structural consistency throughout the study, we employed full-length protein structures predicted by AlphaFold2. While this approach allowed uniform treatment of all targets—including those like JUN and IL-6, whose binding site characteristics are not fully elucidated—it should be noted that it does not integrate available experimental structural data, which might have provided further mechanistic clarity. Moreover, to avoid overlooking potential allosteric sites, we conducted unbiased global docking scans for all targets. Although blind docking is a valuable exploratory tool for identifying novel binding regions, it carries an inherent risk of false positives, particularly for targets with well-defined canonical active sites, such as SERPINE1 and MMP2. This constitutes a recognized limitation of our methodological framework.

Although computational algorithms (such as MCC, MNC, and Degree) aided in target prioritization and helped guide experimental design, they may have overlooked certain biologically relevant targets or pathways. Furthermore, practical constraints—including the limited availability, poor survival, and functional instability of primary alveolar type II (AT2) cells—led us to adopt the A549 cell line as a surrogate model. While this choice improved experimental reproducibility and tractability, it may also limit direct physiological extrapolation to primary cellular environments.

Despite these limitations, our integrative computational and experimental strategy offers a foundational framework for further research. Ongoing work will focus on refining the preclinical profile of resveratrol and assessing its potential for clinical translation in the treatment of fibrosis.

## 5 Conclusion

Collectively, our findings establish resveratrol as a potent inhibitor of alveolar epithelial cell senescence and a promising therapeutic agent capable of ameliorating pulmonary fibrosis, acting potentially through modulation of key aging and senescence-related pathways.

## Data Availability

The original contributions presented in the study are included in the article/Supplementary Material, further inquiries can be directed to the corresponding authors.

## Glossary

ADMET: Absorption, Distribution, Metabolism, Excretion, and Toxicity
AI: Artificial Intelligence
AT1: Alveolar Epithelial Type I Cells
AT2: Alveolar Epithelial Type II Cells
BBB: Blood-Brain Barrier
BLM: Bleomycin
CMC: Carboxymethylcellulose
DEGs: Differentially Expressed Genes
DILI: Drug-Induced Liver Injury
ECM: Extracellular Matrix
FC: Fold Change
GAFF: General Amber Force Field
GEO: Gene Expression Omnibus
GO: Gene Ontology
H&E: Hematoxylin and Eosin Staining
IHC: Immunohistochemistry
IL-6: Interleukin-6
KEGG: Kyoto Encyclopedia of Genes and Genomes
MCC: Maximal Clique Centrality
MD: Molecular Dynamics
MMP2: Matrix Metalloproteinase-2
MNC: Maximum Neighborhood Component
PBS: Phosphate-Buffered Saline
PF: Pulmonary Fibrosis
PK/PD: Pharmacokinetic-Pharmacodynamic
PME: Particle Mesh Ewald
PPB: Plasma Protein Binding
PPI: Protein-Protein Interaction
RES: Resveratrol
Rg: Radius of Gyration
RMSD: Root Mean Square Deviation
RT-qPCR: Real-Time Quantitative PCR
SASPs: Senescence-Associated Secretory Phenotypes
SEM: Standard Error of the Mean
SERPINE1: Serpin Family E Member 1
TCM: Traditional Chinese Medicine
TGF-β1: Transforming Growth Factor Beta 1
α-SMA: Alpha-Smooth Muscle Actin
