# Supplementary material for: Resveratrol attenuates pulmonary fibrosis by inhibiting alveolar epithelial senescence via targeting SASP-related proteins: an integrated bioinformatics-experimental study
Source: Front Pharmacol. 2025 Nov 26;16:1680998. doi: 10.3389/fphar.2025.1680998 (PMC12689916; doi:10.3389/fphar.2025.1680998)
Supplement: Supplementary file 3 [file Supplementaryfile1.docx]

| Properties | Indicator | Resveratrol |
| --- | --- | --- |
| Physicochemical Property | QED | 0.692 |
|  | SAscore | 2 |
|  | Lipinski Rule | Accepted |
|  | Pfizer Rule | Accepted |
|  | GSK Rule | Accepted |
|  | GoldenTriangle | Accepted |
| Absorption | Caco-2 Permeability | -4.917 |
| Distribution | PPB | 88.573 |
|  | BBB | 0.003 |
| Metabolism | CYP2C19 inhibitor | 0.001 |
|  | CYP2C19 substrate | 0 |
|  | HLM Stability | 0.177 |
| Excretion | CLplasma | 9.035 |
|  | T1/2 | 1.456 |
| Toxicity | hERG Blockers | 0.273 |
|  | DILI | 0.062 |
|  | Rat Oral Acute Toxicity | 0.282 |
|  | Drug-induce d Nephrotox icity | 0.074 |
