# Supplementary material for: Resveratrol attenuates pulmonary fibrosis by inhibiting alveolar epithelial senescence via targeting SASP-related proteins: an integrated bioinformatics-experimental study
Source: Front Pharmacol. 2025 Nov 26;16:1680998. doi: 10.3389/fphar.2025.1680998 (PMC12689916; doi:10.3389/fphar.2025.1680998)
Supplement: Supplementary file 10 [file Table7.pdf]

]6O[C@@H](C)[C@H](O)[C@@H](O)[C@H]6O)CC[C@]5(C)[C@H]4CC[C@]3(C)[C@H]1[C@@H]2C

## 1. Physicochemical Property

| Property | Value | Comment |
|---|---|---|
| Molecular Weight | 868.48 | Contain hydrogen atoms. Optimal:100~600 |
| Volume | 847.875 | Van der Waals volume |
| Density | 1.024 | Density = MW / Volume |
| nHA | 16.0 | Number of hydrogen bond acceptors. Optimal:0~12 |
| nHD | 8.0 | Number of hydrogen bond donors. Optimal:0~7 |
| nRot | 7.0 | Number of rotatable bonds. Optimal:0~11 |
| nRing | 9.0 | Number of rings. Optimal:0~6 |
| MaxRing | 20.0 | Number of atoms in the biggest ring. Optimal:0~18 |
| nHet | 16.0 | Number of heteroatoms. Optimal:1~15 |
| fChar | 0.0 | Formal charge. Optimal:-4 ~4 |
| nRig | 48.0 | Number of rigid bonds. Optimal:0~30 |
| Flexibility | 0.146 | Flexibility = nRot /nRig |
| Stereo Centers | 26.0 | Stereo Centers. Optimal: ≤ 2 |
| TPSA | 235.68 | Topological Polar Surface Area. Optimal:0~140 |
| logS | -4.814 | The logarithm of aqueous solubility value. |
| logP | 2.672 | The logarithm of the n-octanol/water distribution coefficients at pH=7.4. |
| logD | 3.48 | The logarithm of the n-octanol/water distribution coefficient. |
| pka (Acid) | 6.874 | Acid-base dissociation constant (pKa) value represents the strength of a drug molecule's acidity or basicity. |
| pka (Base) | 6.449 | Acid-base dissociation constant (pKa) value represents the strength of a drug molecule's acidity or basicity. |
| Melting point | 237.625 | The predicted melting point of a compound is expressed in degrees Celsius (°C).<br>Melting points below 25°C are classified as liquids, while melting points above 25°C are classified as solids. |
| Boiling point | 379.644 | The predicted melting point of a compound is expressed in degrees Celsius (°C).<br>A normal boiling point below 25°C is categorized as a gas. |

## 2. Medicinal Chemistry

| Property | Value | Decision | Comment |
|---|---|---|---|

ADMETlab 3.0

| | | | |
|---|---|---|---|
| QED | 0.168 | 🔴 | ■ A measure of drug-likeness based on the concept of desirability;<br>■ Attractive: > 0.67;<br>■ unattractive: 0.49~0.67;<br>■ too complex: < 0.34 |
| GASA | 1.0 | 🔴 | ■ ES: Easy to synthesize; HS: Hard to synthesize;<br>■ The output value represents the probability of being difficult to synthesize, ranging from 0 to 1. |
| Synth | 6.0 | 🔴 | ■ Synthetic accessibility score is designed to estimate ease of synthesis of drug-like molecules.<br>■ SAscore ≥ 6, difficult to synthesize; SAscore <6, easy to synthesize |
| Fsp3 | 0.956 | 🟢 | ■ The number of sp3 hybridized carbons / total carbon count, correlating with melting point and solubility.<br>■ Fsp$^3$ ≥0.42 is considered a suitable value. |
| MCE-18 | 229.091 | 🟢 | ■ MCE-18 stands for medicinal chemistry evolution.<br>■ MCE-18≥45 is considered a suitable value. |
| NPscore | 2.483 | - | ■ Natural product-likeness score.<br>■ This score is typically in the range from -5 to 5.<br>■ The higher the score is, the higher the probability is that the molecule is a NP. |
| Lipinski Rule | 1.0 | 🔴 | ■ MW ≤ 500; logP ≤ 5; Hacc ≤ 10; Hdon ≤ 5<br>■ If two properties are out of range, a poor absorption or permeability is possible, one is acceptable. |
| Pfizer Rule | 0.0 | 🟢 | ■ logP > 3; TPSA < 75<br>■ Compounds with a high log P (>3) and low TPSA (<75) are likely to be toxic. |
| GSK Rule | 1.0 | 🔴 | ■ MW ≤ 400; logP ≤ 4<br>■ Compounds satisfying the GSK rule may have a more favorable ADMET profile |
| Golden Triangle | 1.0 | 🔴 | ■ 200 ≤ MW ≤ 500; -2 ≤ logD ≤ 5<br>■ Compounds satisfying the Golden Triangle rule may have a more favorable ADMET profile. |
| PAINS | 0 alerts | - | frequent hitters, Alpha-screen artifacts and reactive compound 480 substructures (J Med Chem 201053:2719-40) |
| ALARM NMR | 0 alerts | - | Thiol reactive compounds. |
| BMS | 1 alerts | - | undesirable, reactive compounds 176 substructures (J Chem Inf Model 200646:1060-8) |
| Chelator Rule | 0 alerts | - | Chelating compounds. |
| Colloidal aggregators | 0.784 | - | ■ Category 0: non-colloidal aggregators;<br>■ Category 1: colloidal aggregators.<br>■ The output value is the probability of being colloidal aggregators, within the range of 0 to 1. |

| | | | |
|---|---|---|---|
| FLuc inhibitors | 0.0 | ● | ■ Category 0: non-fLuc inhibitors;<br>■ Category 1: fLuc inhibitors.<br>■ The output value is the probability of being fLuc inhibitors, within the range of 0 to 1. |
| Blue fluorescence | 0.005 | ● | ■ Category 0: non-blue fluorescence;<br>■ Category 1: blue fluorescence.<br>■ The output value is the probability of being blue fluorescence, within the range of 0 to 1. |
| Green fluorescence | 0.0 | ● | ■ Category 0: non-green fluorescence;<br>■ Category 1: green fluorescence.<br>■ The output value is the probability of being green fluorescence, within the range of 0 to 1. |
| Reactive compounds | 0.237 | ● | ■ Category 0: non-reactive compound;<br>■ Category 1: reactive compound.<br>■ The output value is the probability of being reactive compound, within the range of 0 to 1. |
| Promiscuous compounds | 0.015 | ● | ■ Category 0: non-promiscuous compound;<br>■ Category 1: promiscuous compound.<br>■ The output value is the probability of being promiscuous compound, within the range of 0 to 1. |

## 3. Absorption

| Property | Value | Decision | Comment |
|---|---|---|---|
| Caco-2 Permeability | -6.128 | ● | Optimal: higher than -5.15 Log unit |
| MDCK Permeability | -5.029 | ● | ■ low permeability: < 2 × 10-6 cm/s<br>■ medium permeability: 2-20 × $10^{-6}$ cm/s<br>■ high passive permeability: > 20 × $10^{-6}$ cm/s |
| PAMPA | 0.998 | ● | ■ The experimental data for Peff was logarithmically transformed (logPeff).<br>■ Molecules with log Peff values below 2.0 were classified as low-permeability (Category 0), while those with log Peff values exceeding 2.5 were classified as high-permeability (Category 1). |
| Pgp-inhibitor | 0.0 | ● | ■ Category 1: Inhibitor;<br>■ Category 0: Non-inhibitor;<br>■ The output value is the probability of being Pgp-inhibitor |
| Pgp-substrate | 0.882 | ● | ■ Category 1: substrate;<br>■ Category 0: Non-substrate;<br>■ The output value is the probability of being Pgp-substrate |
| HIA | 0.003 | ● | ■ Human Intestinal Absorption<br>■ Category 1: HIA+( HIA < 30%);<br>■ Category 0: HIA-( HIA >= 30%);<br>■ The output value is the probability of being HIA+ |

ADMETlab 3.0

| | | | |
|---|---|---|---|
| $F_{20\%}$ | 0.125 | 🟢 | ■ 20% Bioavailability<br>■ Category 1: F 20% + (bioavailability < 20%);<br>■ Category 0: F 20% - (bioavailability ≥ 20%);<br>■ The output value is the probability of being F 20% + |
| $F_{30\%}$ | 0.749 | 🔴 | ■ 30% Bioavailability<br>■ Category 1: F 30% + (bioavailability < 30%);<br>■ Category 0: F 30% - (bioavailability ≥ 30%);<br>■ The output value is the probability of being F 30% + |
| $F_{50\%}$ | 0.999 | 🔴 | ■ 50% Bioavailability<br>■ Category 1: F 50% + (bioavailability < 50%);<br>■ Category 0: F 50% - (bioavailability ≥ 50%);<br>■ The output value is the probability of being F 50% + |

## 4. Distribution

| Property | Value | Decision | Comment |
|---|---|---|---|
| PPB | 67.018 | 🟢 | ■ Plasma Protein Binding<br>Optimal: < 90%.<br>■ Drugs with high protein-bound may have a low therapeutic index. |
| VDss | -0.381 | 🔴 | ■ Volume Distribution<br>■ Optimal: 0.04-20L/kg |
| BBB | 0.001 | 🟢 | ■ Blood-Brain Barrier Penetration<br>■ Category 1: BBB+; Category 0: BBB-;<br>■ The output value is the probability of being BBB+ |
| Fu | 25.875 | 🟢 | ■ The fraction unbound in plasms<br>■ Low: <5%; Middle: 5~20%; High: > 20% |
| OATP1B1 inhibitor | 1.0 | 🔴 | ■ Category 0: Non-inhibitor; Category 1: inhibitor.<br>■ The output value is the probability of being inhibitor, within the range of 0 to 1. |
| OATP1B3 inhibitor | 1.0 | 🔴 | ■ Category 0: Non-inhibitor; Category 1: inhibitor.<br>■ The output value is the probability of being inhibitor, within the range of 0 to 1. |
| BCRP inhibitor | 0.405 | 🟠 | ■ Category 0: Non-inhibitor; Category 1: inhibitor.<br>■ The output value is the probability of being inhibitor, within the range of 0 to 1. |
| MRP1 inhibitor | 0.022 | 🟢 | ■ Category 0: Non-inhibitor; Category 1: inhibitor.<br>■ The output value is the probability of being inhibitor, within the range of 0 to 1. |

## 5. Metabolism

| Property | Value | Decision | Comment |
|---|---|---|---|
| CYP1A2 inhibitor | 0.0 | 🟢 | ■ Category 1: Inhibitor; Category 0: Non-inhibitor;<br>■ The output value is the probability of being inhibitor. |

ADMETlab 3.0

| | | | |
|---|---|---|---|
| CYP1A2 substrate | 0.0 | 🟢 | ■ Category 1: Substrate; Category 0: Non-substrate;<br>■ The output value is the probability of being substrate. |
| CYP2C19 inhibitor | 0.0 | 🟢 | ■ Category 1: Inhibitor; Category 0: Non-inhibitor;<br>■ The output value is the probability of being inhibitor. |
| CYP2C19 substrate | 0.001 | 🟢 | ■ Category 1: Substrate; Category 0: Non-substrate;<br>■ The output value is the probability of being substrate. |
| CYP2C9 inhibitor | 0.0 | 🟢 | ■ Category 1: Inhibitor; Category 0: Non-inhibitor;<br>■ The output value is the probability of being inhibitor. |
| CYP2C9 substrate | 0.0 | 🟢 | ■ Category 1: Substrate; Category 0: Non-substrate;<br>■ The output value is the probability of being substrate. |
| CYP2D6 inhibitor | 0.0 | 🟢 | ■ Category 1: Inhibitor; Category 0: Non-inhibitor;<br>■ The output value is the probability of being inhibitor. |
| CYP2D6 substrate | 0.0 | 🟢 | ■ Category 1: Substrate; Category 0: Non-substrate;<br>■ The output value is the probability of being substrate. |
| CYP3A4 inhibitor | 0.0 | 🟢 | ■ Category 1: Inhibitor; Category 0: Non-inhibitor;<br>■ The output value is the probability of being inhibitor. |
| CYP3A4 substrate | 1.0 | 🔴 | ■ Category 1: Substrate; Category 0: Non-substrate;<br>■ The output value is the probability of being substrate. |
| CYP2B6 inhibitor | 1.0 | 🔴 | ■ Category 1: Inhibitor; Category 0: Non-inhibitor;<br>■ The output value is the probability of being inhibitor. |
| CYP2B6 substrate | 0.0 | 🟢 | ■ Category 1: Substrate; Category 0: Non-substrate;<br>■ The output value is the probability of being substrate. |
| CYP2C8 inhibitor | 0.991 | 🔴 | ■ Category 1: Inhibitor; Category 0: Non-inhibitor;<br>■ The output value is the probability of being inhibitor. |
| HLM Stability | 0.867 | 🔴 | ■ human liver microsomal (HLM) stability<br>■ Category 0: stable+ (HLM > 30 min); Category 1: unstable- ( HLM ≤ 30 min). The output value is the probability of human liver microsomal instability, where a value closer to 1 indicates a higher likelihood of instability. The range is between 0 and 1. |

## 6. Excretion

| Property | Value | Decision | Comment |
|---|---|---|---|

ADMETlab 3.0

| | | | |
|---|---|---|---|
| $CL_{plasma}$ | 0.3 | ● | ■ The unit of predicted CLplasma penetration is ml/min/kg. >15 ml/min/kg: high clearance; 5-15 ml/min/kg: moderate clearance; < 5 ml/min/kg: low clearance. |
| $T_{1/2}$ | 3.086 | ● | ■ The unit of predicted T1/2 is hours.<br>■ ultra-short half-life drugs: 1/2 < 1 hour; short half-life drugs: T1/2 between 1-4 hours; intermediate short half-life drugs: T1/2 between 4-8 hours; long half-life drugs: T1/2 > 8 hours. |

## 7. Toxicity

| Property | Value | Decision | Comment |
|---|---|---|---|
| hERG Blockers | 0.069 | ● | ■ Molecules with IC50 ≤10µM or ≥50% inhibition at 10 µM were classified as hERG+ (Category 1),<br>■ while molecules with IC50 >10µM or < 50% inhibition at 10µM were classified as hERG - (Category 0).<br>■ The output value is the probability of being hERG+, within the range of 0 to 1. |
| hERG Blockers (10um) | 0.292 | ● | ■ Molecules with IC50 ≤10 µM are classified as hERG+ (Category 1),<br>■ and molecules with IC50 > 10µM are classified as hERG- (Category 0).<br>■ The output value is the probability of being hERG+, within the range of 0 to 1. |
| DILI | 0.951 | ● | ■ Drug Induced Liver Injury.<br>■ Category 1: drugs with a high risk of DILI;<br>■ Category 0: drugs with no risk of DILI.<br>■ The output value is the probability of being toxic. |
| AMES Muta genicity | 0.801 | ● | ■ AMES Toxicity<br>■ Category 1: Ames positive(+);<br>■ Category 0: Ames negative(-);<br>■ The output value is the probability of being toxic. |
| Rat Oral Acute Toxicity | 0.016 | ● | ■ Rat Oral Acute Toxicity.<br>■ Category 0: low-toxicity, > 500 mg/kg;<br>■ Category 1: high-toxicity; < 500 mg/kg.<br>■ The output value is the probability of being toxic, within the range of 0 to 1. |
| FDAMDD | 0.016 | ● | ■ FDA Maximum (Recommended) Daily Dose.<br>■ Category 1: FDAMDD (+);<br>■ Category 0: FDAMDD (-);<br>The output value is the probability of being positive. |
| Skin Sensiti zation | 1.0 | ● | ■ Category 1: Sensitizer;<br>■ Category 0: Non-sensitizer.<br>■ The output value is the probability of being toxic, within the range of 0 to 1. |
| Carcinogeni city | 0.018 | ● | ■ Category 1: carcinogens;<br>■ Category 0: non-carcinogens;<br>■ The output value is the probability of being toxic. |

ADMETlab 3.0

| | | | |
|---|---|---|---|
| Eye Corrosion | 0.0 | ● | ■ Eye Corrosion<br>■ Category 1: corrosives;<br>Category 0: noncorrosives;<br>■ The output value is the probability of being corrosives. |
| Eye Irritation | 0.0 | ● | ■ Eye Irritation<br>■ Category 1: irritants;<br>Category 0: nonirritants;<br>■ The output value is the probability of being irritants. |
| Respiratory | 0.001 | ● | ■ Category 1: respiratory toxicants;<br>■ Category 0: non-respiratory toxicants.<br>■ The output value is the probability of being toxic, within the range of 0 to 1. |
| Human Hep atotoxicity | 0.265 | ● | ■ Human Hepatotoxicity<br>■ Category 1: H-HT positive(+);<br>■ Category 0: H-HT negative(-);<br>■ The output value is the probability of being toxic. |
| Drug-induce d Nephrotox icity | 0.502 | ● | ■ Category 0: non-nephrotoxic (-);<br>■ Category 1: nephrotoxic (+).<br>■ The output value is the probability of being nephrotoxic (+), within the range of 0 to 1. |
| Ototoxicity | 0.998 | ● | ■ Category 0: non-ototoxicity (-);<br>■ Category 1: ototoxicity (+).<br>■ The output value is the probability of being ototoxicity (+), within the range of 0 to 1. |
| Hematotoxic ity | 0.067 | ● | ■ Category 0: non-hematotoxicity (-);<br>■ Category 1: hematotoxicity (+).<br>■ The output value is the probability of being hematotoxicity (+), within the range of 0 to 1. |
| Genotoxicity | 0.003 | ● | ■ Category 0: non-Genotoxicity (-);<br>■ Category 1: Genotoxicity (+).<br>■ The output value is the probability of being ototoxicity (+), within the range of 0 to 1. |
| RPMI-8226 Immunitoxici ty | 0.345 | ● | ■ Category 0: non-cytotoxicity (-);<br>■ Category 1: cytotoxicity (+).<br>■ The output value is the probability of being ototoxicity (+), within the range of 0 to 1. |
| A549 Cytotoxicity | 0.701 | ● | ■ Category 0: non-cytotoxicity (-);<br>■ Category 1: cytotoxicity (+).<br>■ The output value is the probability of being ototoxicity (+), within the range of 0 to 1. |
| Hek293 Cytotoxicity | 0.8 | ● | ■ Category 0: non-cytotoxicity (-);<br>■ Category 1: cytotoxicity (+).<br>■ The output value is the probability of being ototoxicity (+), within the range of 0 to 1. |
| Drug-induce d Neurotoxi city | 0.0 | ● | ■ Category 0: non-neurotoxic (-);<br>■ Category 1: neurotoxic (+).<br>■ The output value is the probability of being neurotoxic (+), within the range of 0 to 1. |

ADMETlab 3.0

## 8. Environmental toxicity

| Property | Value | Comment |
|---|---|---|
| Bioconcentration Factors | 2.019 | ■ Bioconcentration factors are used for considering secondary poisoning potential and assessing risks to human health via the food chain.<br>■ The unit is –log10[(mg/L)/(1000*MW)] |
| $IGC_{50}$ | 3.594 | ■ Tetrahymena pyriformis 50 percent growth inhibition concentration.<br>■ The unit is –log10[(mg/L)/(1000*MW)] |
| $LC_{50}FM$ | 4.182 | ■ 96-hour fathead minnow 50 percent lethal concentration.<br>■ The unit is –log10[(mg/L)/(1000*MW)] |
| $LC_{50}DM$ | 4.739 | ■ 48-hour daphnia magna 50 percent lethal concentration.<br>■ The unit is –log10[(mg/L)/(1000*MW)] |

## 9. Tox21 pathway

| Property | Value | Decision | Comment |
|---|---|---|---|
| NR-AhR | 0.001 | ● | ■ Aryl hydrocarbon receptor<br>■ Category 1: actives ;<br>■ Category 0: inactives;<br>■ The output value is the probability of being active. |
| NR-AR | 0.02 | ● | ■ Androgen receptor<br>■ Category 1: actives ;<br>■ Category 0: inactives;<br>■ The output value is the probability of being active. |
| NR-AR-LBD | 0.0 | ● | ■ Androgen receptor ligand-binding domain<br>■ Category 1: actives ;<br>■ Category 0: inactives;<br>■ The output value is the probability of being active. |
| NR-Aromatase | 0.0 | ● | ■ Category 1: actives ;<br>■ Category 0: inactives;<br>■ The output value is the probability of being active. |
| NR-ER | 0.281 | ● | ■ Estrogen receptor<br>■ Category 1: actives ;<br>■ Category 0: inactives;<br>■ The output value is the probability of being active. |
| NR-ER-LBD | 0.0 | ● | ■ Estrogen receptor ligand-binding domain<br>■ Category 1: actives ;<br>■ Category 0: inactives;<br>■ The output value is the probability of being active. |
| NR-PPAR-gamma | 0.0 | ● | ■ Peroxisome proliferator-activated receptor gamma<br>■ Category 1: actives ;<br>■ Category 0: inactives;<br>■ The output value is the probability of being active. |
| SR-ARE | 0.001 | ● | ■ Antioxidant response element<br>■ Category 1: actives ;<br>■ Category 0: inactives;<br>■ The output value is the probability of being active. |

ADMETlab 3.0

| | | | |
|---|---|---|---|
| SR-ATAD5 | 0.0 | ● | ■ ATPase family AAA domain-containing protein 5<br>■ Category 1: actives ;<br>■ Category 0: inactives;<br>■ The output value is the probability of being active. |
| SR-HSE | 0.0 | ● | ■ Heat shock factor response element<br>■ Category 1: actives ;<br>■ Category 0: inactives;<br>■ The output value is the probability of being active. |
| SR-MMP | 0.0 | ● | ■ Mitochondrial membrane potential<br>■ Category 1: actives ;<br>■ Category 0: inactives;<br>■ The output value is the probability of being active. |
| SR-p53 | 0.004 | ● | ■ p53, a tumor suppressor protein<br>■ Category 1: actives ;<br>■ Category 0: inactives;<br>■ The output value is the probability of being active. |

## 10. Toxicophore Rules

| Property | Value | Comment |
|---|---|---|
| Acute Toxicity Rule | 0 | ■ 20 substructures;<br>■ acute toxicity during oral administration |
| Genotoxic Carcinogenicity Rule | 0 | ■ 117 substructures;<br>■ carcinogenicity or mutagenicity |
| NonGenotoxic Carcinogenicity Rule | 0 | ■ 23 substructures;<br>■ carcinogenicity through nongenotoxic mechanisms |
| Skin Sensitization Rule | 2 alerts | ■ 155 substructures;<br>■ skin irritation |
| Aquatic Toxicity Rule | 2 alerts | ■ 99 substructures;<br>■ toxicity to liquid(water) |
| NonBiodegradable Rule | 1 alerts | ■ 19 substructures;<br>■ non-biodegradable |
| SureChEMBL Rule | 0 | ■ 164 substructures;<br>■ MedChem unfriendly status |
| FAF-Drugs4 Rule | 1 alerts | 154 toxic substructures from FAF-Drug4 |

ADMETlab 3.0
